# Genome-wide association studies in East Asians identify new loci for waist-hip ratio and waist circumference

**DOI:** 10.1038/srep17958

**Published:** 2016-01-20

**Authors:** Wanqing Wen, Norihiro Kato, Joo-Yeon Hwang, Xingyi Guo, Yasuharu Tabara, Huaixing Li, Rajkumar Dorajoo, Xiaobo Yang, Fuu-Jen Tsai, Shengxu Li, Ying Wu, Tangchun Wu, Soriul Kim, Xiuqing Guo, Jun Liang, Dmitry Shungin, Linda S. Adair, Koichi Akiyama, Matthew Allison, Qiuyin Cai, Li-Ching Chang, Chien-Hsiun Chen, Yuan-Tsong Chen, Yoon Shin Cho, Bo Youl Choi, Yutang Gao, Min Jin Go, Dongfeng Gu, Bok-Ghee Han, Meian He, James E. Hixson, Yanling Hu, Tao Huang, Masato Isono, Keum Ji Jung, Daehee Kang, Young Jin Kim, Yoshikuni Kita, Juyoung Lee, Nanette R. Lee, Jeannette Lee, Yiqin Wang, Jian-Jun Liu, Jirong Long, Sanghoon Moon, Yasuyuki Nakamura, Masahiro Nakatochi, Keizo Ohnaka, Dabeeru Rao, Jiajun Shi, Jae Woong Sull, Aihua Tan, Hirotsugu Ueshima, Chen Wu, Yong-Bing Xiang, Ken Yamamoto, Jie Yao, Xingwang Ye, Mitsuhiro Yokota, Xiaomin Zhang, Yan Zheng, Lu Qi, Jerome I. Rotter, Sun Ha Jee, Dongxin Lin, Karen L. Mohlke, Jiang He, Zengnan Mo, Jer-Yuarn Wu, E. Shyong Tai, Xu Lin, Tetsuro Miki, Bong-Jo Kim, Fumihiko Takeuchi, Wei Zheng, Xiao-Ou Shu

**Affiliations:** 1 Department of Epidemiology, Vanderbilt University School of Medicine, Nashville, Tennessee 37203, USA; 2 Shanghai Cancer Institute, Renji Hospital, Shanghai Jiaotong University School of Medicine, Shanghai, China; 3 Key Laboratory of Nutrition and Metabolism, Institute for Nutritional Sciences, Shanghai Institutes for Biological Sciences, Chinese Academy of Sciences, Shanghai, China; 4 Department of Medicine, Yong Loo Lin School of Medicine, National University of Singapore, Singapore; 5 Saw Swee Hock School of Public Health, National University of Singapore, Singapore; 6 Duke–National University of Singapore Graduate Medical School, Singapore; 7 Genome Institute of Singapore, Agency for Science, Technology and Research, Singapore; 8 National Center for Genome Medicine, Institute of Biomedical Sciences, Academia Sinica, Taipei, Taiwan; 9 School of Chinese Medicine, China Medical University, Taichung, Taiwan; 10 Department of Medical Genetics, China Medical University Hospital, Taichung, Taiwan; 11 Department of Health and Nutrition Biotechnology, Asia University, Taichung, Taiwan; 12 Institute for Translational Genomics and Population Sciences, Department of Pediatrics and Medicine, LABioMed at Harbor-UCLA Medical Center, Torrance, California, USA; 13 Division of Preventive Medicine, Department of Family and Preventive Medicine, University of California, San Diego School of Medicine, San Diego, California, USA; 14 Center for Genome Science, National Institute of Health, Osong Health Technology Administration Complex, Chungcheongbuk-do, Republic of Korea; 15 Department of Biomedical Science, Hallym University, Gangwon-do, Korea; 16 Department of Preventive Medicine, College of Medicine, Hanyang University, Seoul, Korea; 17 Department of Preventive Medicine, Seoul National University College of Medicine, Seoul, Korea; 18 Institute for Health Promotion, Yonsei University, Seodaemun-Gu, Seoul 120-749, Korea; 19 Eulji University, Seongnam City, Gyeonggi-do, Korea; 20 Center for Genomic Medicine, Kyoto University Graduate School of Medicine, Kyoto, Japan; 21 Department of Geriatric Medicine, Ehime University Graduate School of Medicine, Ehime, Japan; 22 Department of Health Science, Shiga University of Medical Science, Shiga, Japan; 23 Center for Epidemiologic Research in Asia, Shiga University of Medical Science, Shiga, Japan; 24 Cardiovascular Epidemiology, Kyoto Women’s University, Kyoto, Japan; 25 Department of Nursing, Tsuruga University of Nursing, Tsuruga, Japan; 26 Department of Clinical Sciences, Genetic & Molecular Epidemiology Unit, Lund University Diabetes Center, Skåne University Hospital, Malmö 205 02, Sweden; 27 Department of Public Health and Clinical Medicine, Unit of Medicine, Umeå University, Umeå 901 87, Sweden; 28 Department of Epidemiology, Tulane University School of Public Health and Tropical Medicine, New Orleans, Louisiana, USA; 29 Division of Biostatistics, Washington University School of Medicine, St. Louis, Missouri, USA; 30 Human Genetics Center, University of Texas School of Public Health, Houston, Texas, USA; 31 Division of Population Genetics, State Key Laboratory of Cardiovascular Disease, Fuwai Hospital, National Center for Cardiovascular Diseases, Chinese Academy of Medical Sciences and Peking Union Medical College, Beijing, China; 32 Center for Genomic and Personalized Medicine, Guangxi Medical University, Nanning, Guangxi, China; 33 Institute of Urology and Nephrology, The First Affiliated Hospital of Guangxi Medical University, Nanning, Guangxi, China; 34 Department of Chemotherapy, The Affiliated Tumor Hospital of Guangxi Medical University, Nanning 530021, Guangxi, China; 35 Medical Scientific Research Center, Guangxi Medical University, Nanning, Guangxi 530021, China; 36 Department of Occupational Health and Environmental Health, School of Public Health, Guangxi Medical University, Nanning, Guangxi, China; 37 Key Laboratory of Environment and Health, Ministry of Education, School of Public Health, Tongji Medical College, Huazhong University of Science and Technology, Wuhan, Hubei, China; 38 State Key Laboratory of Molecular Oncology, Cancer Institute & Hospital, Chinese Academy of Medical Sciences and Peking Union Medical College, Beijing, China; 39 Department of Nutrition, Harvard School of Public Health, Boston, Massachusetts 02115, USA; 40 Department of Gene Diagnostics and Therapeutics, Research Institute, National Center for Global Health and Medicine, Shinjuku-ku, Tokyo 1628655, Japan; 41 Bioinformatics Section, Center for Advanced Medicine and Clinical Research, Nagoya University Hospital, Showa-ku, Nagoya 466-8560, Japan; 42 Department of Geriatric Medicine, Graduate School of Medical Sciences, Kyushu University, Higashi-ku, Fukuoka 812-8582, Japan; 43 Department of Medical Chemistry, Kurume University School of Medicine, Kurume, Fukuoka 830-0011, Japan; 44 Department of Genome Science, Aichi-Gakuin University, School of Dentistry, Chikusa-ku, Nagoya, 464-8651, Japan; 45 Department of Nutrition, University of North Carolina, Chapel Hill, North Carolina 27599, USA; 46 Department of Genetics, University of North Carolina, Chapel Hill, North Carolina 27599, USA; 47 Department of Endocrinology, the Central Hospital of Xuzhou, Affiliated Hospital of Southeast University, Xuzhou, Jiangsu, China; 48 Office of Population Studies Foundation Inc., University of San Carlos, Talamban, Cebu City, Philippines

## Abstract

Sixty genetic loci associated with abdominal obesity, measured by waist circumference (WC) and waist-hip ratio (WHR), have been previously identified, primarily from studies conducted in European-ancestry populations. We conducted a meta-analysis of associations of abdominal obesity with approximately 2.5 million single nucleotide polymorphisms (SNPs) among 53,052 (for WC) and 48,312 (for WHR) individuals of Asian descent, and replicated 33 selected SNPs among 3,762 to 17,110 additional individuals. We identified four novel loci near the *EFEMP1, ADAMTSL3* , *CNPY2*, and *GNAS* genes that were associated with WC after adjustment for body mass index (BMI); two loci near the *NID2* and *HLA-DRB5* genes associated with WHR after adjustment for BMI, and three loci near the *CEP120*, *TSC22D2*, and *SLC22A2* genes associated with WC without adjustment for BMI. Functional enrichment analyses revealed enrichment of corticotropin-releasing hormone signaling, GNRH signaling, and/or CDK5 signaling pathways for those newly-identified loci. Our study provides additional insight on genetic contribution to abdominal obesity.

Abdominal obesity, typically measured by waist circumference (WC) and waist-hip ratio (WHR), is more closely associated with metabolic dysfunctions that are related to cardiovascular diseases than is general obesity^1^, which is generally assessed by body mass index (BMI). Previous studies have identified multiple genetic loci associated with WC and WHR^2^,^3^,^4^,^5^,^6^,^7^,^8^,^9^,^10^,^11^,^12^,^13^. However, the majority of these studies were conducted in populations of European ancestry or included a limited number of East Asians^9^. East Asians tend to have a higher level of abdominal fat, despite relatively low BMI values; and experience a higher metabolic disease risk than European-ancestry individuals with the same BMI level^14^. Therefore, it is particularly important to investigate the genetic determinants of abdominal fat, i.e. WC and WHR, in East Asian populations.

We previously reported genetic loci for BMI using data from the Asian Genetic Epidemiology Network (AGEN) Consortium^15^,^16^. In this study, we conducted meta-analyses of data from genome-wide association studies (GWAS) of WC and WHR to identify new genetic loci and evaluate associations of previously-identified genetic loci with overall and abdominal obesity in our study populations.

## Results

Our initial meta-analysis used two complementary but related measures of abdominal obesity, WC and WHR, as the outcome variables, and analyzed the association of WC and WHR with approximately 2.5 million genotyped or imputed SNPs as well as about 50,000 typed exome-chip variants. The total sample sizes in Stage I were 53,052 for WC and 48,312 for WHR. We selected 33 SNPs at 33 independent loci with *P* < 1.00 × 10^−6^, based on the GWAS data that were recruited at the first round of Stage I, for a *de novo* replication (Stage II) of associations with WC or WHR. The replication genotyping was conducted at three study sites (see Supplementary Table 3 online) comprising 3,762 to 17,110 Asian-ancestry individuals based on availability of *de novo* data for each SNP. Participating studies are described in the Supplementary Information and Supplementary Tables 1 to 3 online.

**Figure 1:**
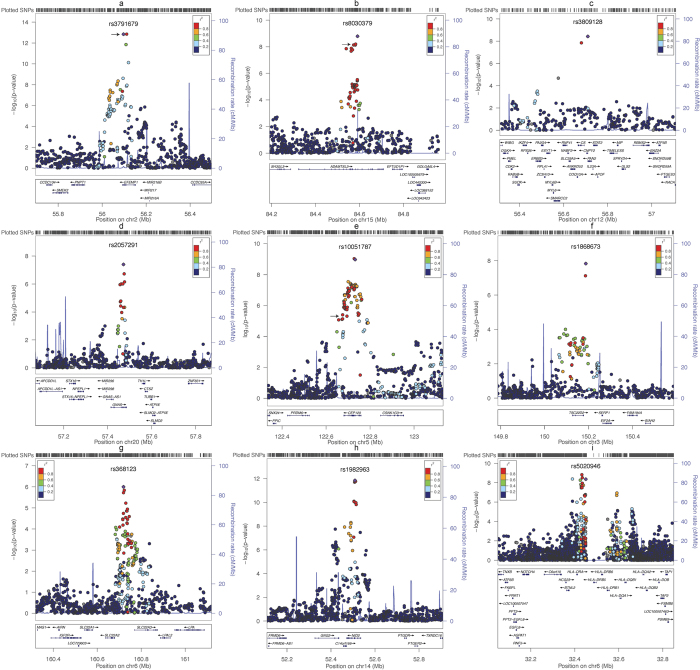
Regional plots for the newly-identified loci in this study. SNPs are plotted by their position on the chromosome against their association (−log10 P value) with the trait of interest (as shown in Table 1) using stage I (GWAS meta-analysis) data. Estimated recombination rates (from HapMap) are plotted in cyan to reflect the local LD structure. The SNPs surrounding the top SNP are color-coded (see inset) to reflect their LD with the top SNP (using pair-wise r2 values from HapMap CHB + JPT data). Genes and positions of exons, as well as directions of transcription, are shown below the plots (using data from the UCSC Genome Browser, genome.ucsc.edu). The arrows shown in Plots a, b, e indicate SNPs that were previously reported to be associated with height. Plots were generated using LocusZoom.

The associations of SNPs with WC or WHR were analyzed with or without adjustment for BMI (see Methods), following the common practice employed in published studies^2^,^3^,^4^,^5^,^6^,^7^,^8^,^9^,^10^,^11^,^12^,^13^. Thus, there were four traits included in this study: WC with adjustment for BMI (WCadjBMI), WHR with adjustment for BMI (WHRadjBMI), WC without adjustment for BMI (WCnoBMI), and WHR without adjustment for BMI (WHRnoBMI). The results of the initial Stage I and Stage II for the selected 33 SNPs are presented in Supplementary Table 4 online. In Table 1 (see also Fig. 1 and Supplementary Table 5), we present the newly-identified loci that were associated with WCadjBMI, WHRadjBMI, and WCnoBMI at a genome-wide significance level (*P* < 5.00 × 10^−8^) based on Stage I data alone or the combined Stage I and Stage II data in Asian-ancestry populations. For WCadjBMI, we have identified four new loci (index SNPs) near these genes: *EFEMP1* (rs3791679, *P* = 4.86 × 10^−14^), *ADAMTSL3* (rs8030379, *P* = 1.62 × 10^−9^), *CNPY2* (rs3809128, *P* = 3.74 × 10^−9^), and *GNAS* (rs2057291, *P* = 4.02 × 10^−8^); for WHRadjBMI, we have identified two loci near the *NID2* (rs1982963, *P* = 1.07 × 10^−14^) and *HLA-DRB5* (rs5020946, *P* = 1.30 × 10^−9^) genes; for WCnoBMI, we have identified three loci near the *CEP120* (rs10051787, *P* = 7.23 × 10^−12^), *TSC22D2* (rs1868673, *P* = 1.49 × 10^−8^), and *SLC22A2* (rs368123, *P* = 2.64 × 10^−8^) genes. In addition, three SNPs near three genes (*ADAMTS3, IHH, QSOX2*) for WCadjBMI, and two SNPs near two genes (*PPAP2B, PACSIN3*) for WHRadjBMI were found to approach the genome-wide significance level (*P* < 7.56 × 10^−7^) (see Supplementary Tables 5,6,7 online). We requested an *in silico* replication for the 14 SNPs described above in the Genetic Investigation of ANthropometric Traits (GIANT) consortium^13^. No data were available for rs3809128 (MAF < 0.01 in CEU) near *CNPY2* in the GIANT data. As shown in Supplementary Table 6 online, the association directions were consistent for 12 out of the remaining 13 SNPs (*P* = 0.0034 by the binomial test), although the explained variances were generally smaller than those observed in East Asians (see Supplementary Table 5 online). The SNP rs2057291 near *GNAS* exhibited an opposite association direction in the GIANT data. Seven of the ten loci for WCadjBMI and WHRadjBMI were oppositely associated with BMI, but all three WCnoBMI loci had a consistent association direction with BMI in the GIANT data. In this study, we found no genetic association with WHRnoBMI at loci other than those previously reported (see Supplementary Table 8 online). The variation explained by each newly-identified SNP ranged from 0.02% to 0.09% (Table 1, and Supplementary Table 5 online). There were no Stage II replication data for some of the loci because they were identified after the second round of Stage I GWAS data were added to the meta-analysis due to the expansion of the AGEN, which occurred after the original 33 replication SNPs were selected.

Additional analyses examined effect sizes for differences across sex and population. Analyses stratified by sex (Table 2, and Supplementary Table 5 online) revealed that association of rs3791679 near the *EFEMP1* gene with WCadjBMI was significantly stronger among men than among women (effect size: 4.04 vs 2.43, *P* for homogeneity test = 0.04), and association of rs1982963 near the *NID2* gene with WHRadjBMI was significantly weaker among men than among women (effect size: 2.88 vs 6.26, *P* for homogeneity test = 0.009). No significant heterogeneity across populations of Chinese, Korean, Japanese, or Filipino was found for the newly-identified loci (data not shown).

Supplementary Table 7 online shows the association of the newly-identified loci with different obesity-related traits. The three novel loci (*CEP120, TSC22D2*, and *SLC22A2*) for WCnoBMI were much less significantly associated with WCadjBMI; the newly-identified loci for WCadjBMI or WHRadjBMI were either unassociated with BMI (*EFEMP1, CNPY2, GNAS*, and *HLA-DRB5*) or negatively associated with BMI (*ADAMTSL3* and *NID2*). Of these 11 loci for WCadjBMI and WHRadjBMI, ten had an opposite association direction with BMI. Using the Wald test of whether the BMI-adjusted effect was equal to its expectation proposed by Aschard *et al.*^17^, we found Bonferroni-corrected significant p-values (0.05/11) for rs11103390 at *QSOX2* (WCadjBMI) and rs1982963 at *NID2* (WHRadjBMI), suggesting that the associations of these two SNPs with WCadjBMI or WHRadjBMI may have been influenced by their direct genetic association with BMI. The newly-identified seven loci for WCadjBMI were all moderately or strongly associated with height and the three novel loci for WCnoBMI were also moderately associated with height. None of 14 newly-identified loci were associated with diabetes at P < 0.004 (0.05/14).

Previous studies have reported about 60 genetic loci associated with abdominal obesity^2^,^3^,^4^,^5^,^6^,^7^,^8^,^9^,^10^,^11^,^12^,^13^ and about 100 genetic loci associated with overall obesity^15^,^16^,^18^, with the majority of those loci being identified in populations of European ancestry. In those studies, the reported associations with WHR were generally adjusted for BMI, while the reported associations with WC were not adjusted for BMI in most studies. Due to the close correlation (r = 0.83 based on data from the Shanghai genome-wide association studies (SGWAS)) between WC and BMI, there was substantial overlap between loci that were associated with WC and BMI. In the current study, we evaluated the associations of those reported loci with WCadjBMI, WHRadjBMI, WCnoBMI, and WHRnoBMI. The associations with at least one trait that achieved a Bonferroni-corrected significance level (*P* < 0.05/60 ≈1.0 × 10^−3^) and the associations by sex are shown in Supplementary Tables 8 to 11 online (for WCadjBMI, WHRadjBMI, WCnoBMI, and WHRnoBMI, respectively). We found that 23 previously-reported loci for abdominal obesity were significantly associated with WCadjBMI (see Supplementary Table 11 online) and/or WHRadjBMI (see Supplementary Table 10 online) and 18 previously-reported BMI/WC loci were significantly associated with WCnoBMI, among men or women or both at *P* < 1.0 × 10^−3^ (see Supplementary Table 9 online). Of note, 17 of those 18 loci associated with WCnoBMI were not significantly associated with WCadjBMI. The only SNP demonstrating significant association with WCadjBMI was rs12229654, at our previously-identified Asian-specific BMI locus *MYL2*. Consistent with previous findings^7^,^19^, we observed that 10 out of 23 replicated genetic loci for abdominal obesity showed significant sex differences (*P* for homogeneity test  < 0.05), 9 of which showed larger effects in women than men (see Supplementary Table 10 online). In contrast, 4 replicated WCnoBMI loci revealed significant sex differences, 3 of which showed larger effects in men (see Supplementary Table 9 online). It is worth noting that the SNP rs12229654 at *MYL2* and its related SNP rs671 at *ALDH2* were associated with every obesity trait analyzed (WCadjBMI, WHRadjBMI, WCnoBMI, and WHRnoBMI), with larger effects observed in men.

We examined the modification effect of alcohol consumption on the association between the two SNPs in the 12q24 region^16^ (rs12229654 and rs671) and WC/WHR using data from the SGWAS, for which we had direct access to individual data. We found that the effects of the SNP rs12229654 on WC and WHR, with or without adjustment for BMI, were mainly observed among non-drinkers (Supplementary Table 13 online). The sex-difference of rs671 effect on WC and WHR is less evident.

We evaluated putative functional significance for each newly-identified locus using the Encyclopedia of DNA Elements (ENCODE) and expression quantitative trait locus (eQTL) databases^20^. Three of these novel SNPs, rs3809128 near *CNPY2*, rs2057291 at *GNAS*, and rs3791679 near *EFEMP1*, were predicted to be functional as they are located either in promoter or enhancer regions based on epigenomic data from the ENCODE project. The ChromHMM annotation on nine ENCODE cell lines have revealed that the SNP rs3809128 resides in an active promoter of the nearest gene, canopy FGF signaling regulator 2 (*CNPY2*). The DNase and ChIP-Seq has revealed that the SNP rs3809128 is present in the DNase I hypersensitive site (DHS) and in the binding regions of multiple transcription factors (TFs). In particular, this variant has been shown to be associated with the expression of *CNYP2* based on a previous eQTL study^19^. The SNP rs2057291 is located in intron 2 of the gene *GNAS*. A search of RegulomeDB^21^ indicates that this variant is annotated to the TF SRF predicted motif. This variant was also observed to be present in the DHS and multiple TFs peaks. The SNP rs3791679 is located in the first intron of the gene *EFEMP1* and resides in an enhancer region. According to RegulomeDB annotation, the SNP rs3791679 lies in the TF POU3F2 predicted motif. In addition, the DHS and the TF STAT3 peak were found to harbor the variant. Other potential functional variants which are in strong linkage disequilibrium (LD)(r^2^ > 0.6) with the newly-identified SNPs are listed in Supplementary Table 12 online.

We conducted two separate functional enrichment analyses for genes located near the newly- and previously-identified loci for WC or WHR, one for the loci associated with WCadjBMI and/or WHRadjBMI, another for the loci associated with WCnoBMI. The corticotropin-releasing hormone signaling (*P* = 5.72 × 10^−4^) pathway and Gonadotropin Releasing Hormone (GNRH) signaling (*P* = 8.63 × 10^−4^) pathway, were found to be the most significantly enriched for the loci associated with WCadjBMI and/or WHRadjBMI; and the CDK5 signaling (*P* = 1.66 × 10^−4^) and corticotropin-releasing hormone signaling (*P* = 2.21 × 10^−4^) pathway were significantly enriched for the loci associated with WCnoBMI.

## Discussion

Previously-reported genetic loci associated with WC, mainly from studies conducted in European-ancestry populations, were generally not adjusted for BMI. There was substantial overlapping between loci that were associated with WC and BMI due to high correlation between those two measurements. Three novel SNPs (rs10051787 near *CEP120*, rs1868673 near *TSC22D2*, and rs368123 at *SLC22A2*), newly identified for WCnoBMI in this study, did not reach the genome-wide significance level in our previous meta-analysis for BMI, and thus were not identified as the BMI loci. After adjustment for BMI, the association of these three SNPs with WCadjBMI was substantially attenuated. In addition, our study showed that previously-reported genetic loci for WC or BMI were generally not significantly associated with WCadjBMI (see Supplementary Table 11 online), suggesting that these two highly-correlated anthropometrics capture a similar biological phenotype. On the other hand, the genetic loci associated with WCadjBMI and/or WHRadjBMI were not typically associated with BMI or showed an opposite association direction (see Supplementary Table 7 and Supplementary Table 14 online). These findings reveal differences in genetic predisposition to overall as opposed to abdominal obesity, as well as the genetic regulation of body fat distribution versus BMI.

Two newly-identified WCadjBMI-related loci (*EFEMP1, ADAMTSL3*) and the newly-identified WCnoBMI-related loci (*CEP120*) were previously reported to affect height as well^11^,^22^, suggesting these loci may be related to general body frame size. The SNP rs3791679 near *EFEMP1* seems particularly relevant to WC and height. *EFEMP1* encodes an extracellular matrix protein containing tandemly-repeated epidermal growth factor-like repeats, which are able to stimulate DNA synthesis and are involved in cell proliferation^23^. *EFEMP1* knockout mice exhibited reduced reproductivity, and displayed an early onset of aging-associated phenotypes including reduced lifespan and decreased body mass^24^. A recent study showed that the *EFEMP1* locus affected growth rate in children^25^.

Another newly-identified WCnoBMI-related SNP, rs1868673, resides near the gene *TSC22D2*. A recent study indicated that this locus was associated with circulating levels of adiponectin, a hormone produced predominantly by adipocytes^26^.

We previously reported the association of two related SNPs in the 12q24 region (rs12229654 at gene *MYL2* and rs671 at *ALDH2*, r^2^ = 0.58)^15^,^16^, a specific polymorphic region for East Asian-ancestry populations, with BMI. This association was significantly stronger among men than among women. We observed similar associations of those SNPs with WC and WHR, with or without adjustment for BMI (Supplementary Tables 8 to 11 online). The sex differences of associations were more prominent for WHR. The SNPs rs12229654 and rs671 have been reported to be associated with HDL cholesterol^27^, levels of gamma glutamyl transpeptidase^27^, elevated blood pressure^28^, lower risk of coronary heart disease^29^ and alcohol consumption^30^ in Asian-ancestry populations. Our previous study^16^ suggested an antagonistic effect of alcohol consumption on the *ALDH2*-BMI association. The ALDH2*1 BMI-increasing effect was mainly observed among non-drinkers. In this study, we found that the effects of the SNP rs12229654 at *MYL2* on WC and WHR, with or without adjustment for BMI, were mainly seen among non-drinkers (Supplementary Table 13 online).

Consistent with previous reports from European populations, we found evidence for multiple loci with significant sex differences for abdominal obesity in East Asians, with a generally more prominent effect in women, although larger effects in men than in women were observed for loci *EFEMP1*, *MYL2/ALDH2*, and *FGFR4*. Typically, men have more visceral fat, whereas women have more subcutaneous fat. It is well known that sex hormones play an important role in the regulation of body fat distribution^31^, but the underlying genetic mechanisms remain unclear. It would be worthwhile to investigate the association between these genetic loci and sex hormone levels.

The corticotropin-releasing hormone signaling (*P* = 5.72 × 10^−4^) pathway and Gonadotropin Releasing Hormone (GNRH) signaling (*P* = 8.63 × 10^−4^) pathway were found to be significantly enriched for genes associated with WCadjBMI and/or WHRadjBMI loci in this study. The corticotropin-releasing hormone signaling (*P* = 5.72 × 10^−4^) pathway was found to be related to BMI in our previous report for BMI loci^16^. Corticotrophin-releasing hormone causes release of adrenocorticotropic hormone from the pituitary gland. Its main role in the body is as the central driver of the stress hormone system, known as the hypothalamic-pituitary-adrenal axis. Gonadotrophin-releasing hormone plays a key role in coordinating the levels of hormones in the hypothalamic-pituitary-gonadal axis; these hormones act on the testes and ovaries to initiate and maintain their reproductive functions. These results provide additional evidence affirming the involvement of stress and sex hormones in obesity and fat distribution.

Recently, Aschard *et al.*^17^ raised a concern that adjusting for BMI may bias genetic effects on WC/WHR and observed enrichment of SNPs that were associated with WC/WHR and BMI in the opposite directions, as shown in the Heid *et al.* study^7^ and in our study (see Supplementary Table 5 online). As shown in the results section, the associations of SNPs rs11103390 at *QSOX2* with WCadjBMI or rs1982963 at *NID2* with WHRadjBMI may be influenced by their direct genetic association with BMI. Further studies are warranted to evaluate this influence.

Another limitation of this study is lack of replication data for some SNPs identified in the expanded Stage I study which will need to be confirmed in future studies.

In conclusion, our study identified at the genome-wide significance level four novel loci near the *EFEMP1*, *ADAMTSL3, CNPY2*, and *GNAS* genes that are associated with WCadjBMI, two loci near the *NID2* and *HLA-DRB5* genes that were associated with WHRadjBMI, and three novel loci near the *CEP120*, *TSC22D2*, and *SLC22A2* genes that were associated with WCnoBMI. Of about 60 genetic loci previously identified for abdominal obesity in predominantly European populations, a similar association was found in our study for 23 in East Asians, suggesting Asian- and European-ancestry individuals have both a shared and a unique genetic basis for abdominal obesity. Functional analyses suggest that genetic regulation for abdominal fat distribution may occur via the corticotropin-releasing hormone signaling, GNRH signaling, and/or CDK5 signaling pathway.

## Materials and Methods

### Study design

This study had two stages. Stage I was a meta-analysis of study-specific results on the association between SNPs and WC or WHR from GWAS that participated in the Asian Genetic Epidemiology Network (AGEN) Consortium. Stage II conducted *de novo* or *in silico* replication analyses to further examine the association for some promising SNPs selected from the Stage I meta-analysis. Supplementary Tables 1,2,3 online and the Supplementary Information page online summarize the basic data for all participating studies.

### Stage I samples and genotyping

In stage I, the participating GWAS were recruited in two rounds because of the expansion of the AGEN. The first round included eight GWAS with a total 27,537 (for WC) or 25,241 (for WHR) individuals of East Asian ancestry, and the second round included eight more GWAS with a total 17,072 (for WC) or 14,628 (for WHR) individuals of East Asian ancestry. In addition, Stage I analysis included about 50,000 SNPs with minor allele frequency over 1% from 8,443 subjects with genotyping data by exome-chip. Therefore, the total sample sizes in Stage I were 53,052 for WC and 48,312 for WHR.

The sample sizes of the 16 participating GWAS in Stage I varied from 695 to 9,279, comprising a total of 44,596 individuals. Nine studies used Affymetrix arrays, and seven studies used the Illumina platform (detailed information is provided in the Supplementary Information online). To allow for combination of the data derived from different genotyping platforms and to improve coverage of the genome, genotype imputation was performed by each participating study using either MACH or IMPUTE with HapMap CHB+JPT data (release #22, build 36) as the imputation reference panel (see Supplementary Table 2 online).

### Stage I statistical analysis

A uniform statistical analysis protocol was followed by each participating study. To improve the normality of the WC and WHR distribution and alleviate the impact of outliers, rank-based inverse normal transformation (INT) was applied to WC and WHR data separately for each sex by each study. INT involves ranking all WC and WHR values, transforming these ranks into quantiles and, finally, converting the resulting quantiles into normal deviates. Associations between SNPs and the inverse normal-transformed WC and WHR were analyzed with a linear regression model, assuming an underlying additive genetic model and adjusting for age (continuous), age-squared, and sex (if applicable). Stratified analyses by sex were also performed for each study. To evaluate the genetic influence on body fat distribution, all the analyses were conducted using two separate models, one with adjustment for BMI, another without adjustment for BMI.

Next, we carried out meta-analyses using a weighted average method with inverse-variance weights. The meta-analyses were carried out on all data combined and also stratified by sex using the freely available METAL software. The presence of heterogeneity across studies and between sexes was tested with Cochran’s Q statistics^32^.

To correct each study for residual population stratification or cryptic relatedness, the meta-analyses were performed with genomic control correction^33^ by adjusting for the study-specific inflation factor (λ), which ranged from 1.000 to 1.078 in Stage I (see Supplementary Table 2 online). After this adjustment, the estimated inflation factors for the Stage I meta-analysis statistic were 1.053 (WCadjBMI), 1.042 (WHRadjBMI), 1.091 (WCnoBMI), and 1.054 (WHRnoBMI), which were further adjusted for when calculating the Stage I results.

### Stage II replication analysis

We selected 33 SNPs at 33 independent loci with *P* < 1.00 × 10^−6^ for the associations with WC or WHR for a *de novo* replication (Stage II), based on the GWAS data that were recruited in the first round of Stage I. The replication genotyping was conducted at three study sites (see Supplementary Table 1 online) comprising 3,762 to 17,110 Asian-ancestry individuals based on availability of *de novo* data for each SNP. Participating studies are described in the Supplementary Information and Supplementary Tables 1 to 3 online.

Each study individually conducted a similar analysis of the association of WC and WHR with the selected SNPs, using the same protocol used in Stage I. The Stage II data were combined using the same meta-analysis methods as in Stage I. Finally, we used meta-analysis to combine all data from both Stages I and II.

## Additional Information

**How to cite this article**: Wen, W. *et al.* Genome-wide association studies in East Asians identify new loci for waist-hip ratio and waist circumference. *Sci. Rep.* **6**, 17958; doi: 10.1038/srep17958 (2016).

## Supplementary Material

Supplementary Information

Supplementary Table S1

Supplementary Table S2

Supplementary Table S3

Supplementary Table S4

Supplementary Table S5

Supplementary Table S6

Supplementary Table S7

Supplementary Table S8

Supplementary Table S9

Supplementary Table S10

Supplementary Table S11

Supplementary Table S12

Supplementary Table S13

Supplementary Table S14

## Figures and Tables

**Table 1:** Newly identified loci associated with waist circumference (WC)/WHR variation in Asian-ancestry populations.

Nearby gene	Chr	SNP	Alleles^a^	EAF^b^	StageI*P*	Stage II *P*	Stage I & II			EV(%)^e^
							Number	*β* (SE)^c^	*P*^d^	
WCadjBMI										
EFEMP1	2	rs3791679	A/G	0.21	1.43E-13	3.63E-02	64454	2.87(0.38)	4.86E-14	0.03%
ADAMTSL3	15	rs8030379	A/G	0.76	1.62E-09	NA	50668	2.46(0.41)^f^	1.62E-09	0.02%
CNPY2	12	rs3809128	C/T	0.80	3.74E-09	NA	30368	3.69(0.63)^f^	3.74E-09	0.04%
GNAS	20	rs2057291	G/A	0.73	4.02E-08	NA	38613	2.52(0.46)^f^	4.02E-08	0.02%
WHRadjBMI										
NID2	14	rs1982963	A/G	0.81	1.41E-12	1.71E-03	56208	4.82(0.62)	1.07E-14	0.07%
HLA-DRB5	6	rs5020946	T/G	0.41	4.38E-09	1.13E-01	49519	3.15(0.52)	1.30E-09	0.05%
WCnoBMI										
CEP120	5	rs10051787	T/C	0.40	1.07E-09	1.40E-03	60909	3.96(0.58)	7.23E-12	0.08%
TSC22D2	3	rs1868673	C/A	0.48	1.49E-08	NA	36247	4.36(0.77)^f^	1.49E-08	0.09%
SLC22A2	6	rs368123	G/A	0.39	1.00E-06	7.29E-03	62430	3.16(0.57)	2.64E-08	0.05%

^a^Shown as: effect allele/other allele.

^b^Effect allele frequency in Asian-ancestry populations, estimated from stage I studies.

^c^Per allele effect of SNPs in percentile of standard deviation, derived from meta-analysis.

^d^Derived from meta-analysis. The *P* values for the combined data were adjusted for both study-specific inflation factors and the estimated inflation factor for the stage I meta-analysis statistic.

^e^Explained variance, estimated from combined stage I and II data.

^f^Stage I results are shown.

**Table 2:** Newly identified loci associated with WC/WHR variation in East Asian-ancestry populations, by gender.

Nearby gene	Chr	SNP	Alleles	Among men			Among women			Test for homogeneity
				Number	β(SE)	P	Number	β(SE)	P	P
WCadjBMI										
EFEMP1	2	rs3791679	A/G	21172	4.04(0.63)	1.76E-10	42605	2.43(0.49)	6.31E-07	4.37E-02
ADAMTSL3	15	rs8030379	A/G	16135	1.41(0.72)	4.89E-02	33848	2.37(0.52)	5.70E-06	2.80E-01
CNPY	12	rs3809128	C/T	11098	2.08(1.07)	5.15E-02	18575	1.59(0.88)	7.30E-02	7.24E-01
GNAS	20	rs2057291	G/A	11626	3.10(1.01)	2.15E-03	26302	1.99(0.60)	8.70E-04	3.44E-01
WHRadjBMI										
NID2	14	rs1982963	A/G	17516	2.88(1.04)	5.37E-03	33264	6.26(0.78)	8.88E-16	9.32E-03
HLA-DRB5	6	rs5020946	T/G	14055	3.17(0.97)	1.10E-03	30049	3.57(0.65)	3.54E-08	7.32E-01
WCnoBMI										
CEP120	5	rs10051787	T/C	20191	4.60(1.00)	4.15E-06	40033	3.82(0.70)	4.89E-08	5.27E-01
TSC22D2	3	rs1868673	C/A	10742	5.58(1.54)	2.93E-04	24820	4.28(0.91)	2.40E-06	4.67E-01
SLC22A2	6	rs368123	G/A	21164	2.30(0.97)	1.73E-02	40581	3.64(0.70)	1.91E-07	2.61E-01

Alleles: Shown as effect allele/other allele. β: Effect of SNPs per allele in percentile of standard deviation, derived from meta-analysis. P: Derived from meta-analysis adjusted for both study-specific inflation factors (for stage I, II) and the estimated inflation factor for the stage I meta-analysis statistic.

## References

[b1] Ritchie S. A. & Connell J. M. The link between abdominal obesity, metabolic syndrome and cardiovascular disease. NutrMetab Cardiovasc. 17, 319–326 (2007). 10.1016/j.numecd.2006.07.005 17110092

[b2] Fox C. S. *et al.* Genome-wide association to body mass index and waist circumference: the Framingham Heart Study 100K project. BMC.Med.Genet. 8 Suppl 1, S18 (2007). 17903300 10.1186/1471-2350-8-S1-S18 PMC1995618

[b3] Chambers J. C. *et al.* Common genetic variation near MC4R is associated with waist circumference and insulin resistance. Nat.Genet. 40, 716–718 (2008). 18454146 10.1038/ng.156

[b4] Lindgren C. M. *et al.* Genome-wide association scan meta-analysis identifies three Loci influencing adiposity and fat distribution. PLoS.Genet. 5, e1000508 (2009). 19557161 10.1371/journal.pgen.1000508 PMC2695778

[b5] Heard-Costa N. L. *et al.* NRXN3 is a novel locus for waist circumference: a genome-wide association study from the CHARGE Consortium. PLoS.Genet. 5, e1000539 (2009). 19557197 10.1371/journal.pgen.1000539 PMC2695005

[b6] Speliotes E. K. *et al.* Association analyses of 249,796 individuals reveal 18 new loci associated with body mass index. Nat.Genet. (2010). 10.1038/ng.686. PMC3014648 20935630

[b7] Heid I. M. *et al.* Meta-analysis identifies 13 new loci associated with waist-hip ratio and reveals sexual dimorphism in the genetic basis of fat distribution. Nat.Genet. 42, 949–960 (2010). 20935629 10.1038/ng.685 PMC3000924

[b8] Kraja A. T. *et al.* A bivariate genome-wide approach to metabolic syndrome: STAMPEED consortium. Diabetes 60, 1329–1339 (2011). 21386085 10.2337/db10-1011 PMC3064107

[b9] Cho Y. S. *et al.* A large-scale genome-wide association study of Asian populations uncovers genetic factors influencing eight quantitative traits. Nat.Genet. 41, 527–534 (2009). 19396169 10.1038/ng.357

[b10] Croteau-Chonka D. C. *et al.* Genome-wide association study of anthropometric traits and evidence of interactions with age and study year in Filipino women. Obesity.(Silver.Spring) 19, 1019–1027 (2011). 20966902 10.1038/oby.2010.256 PMC3046220

[b11] Berndt S. I. *et al.* Genome-wide meta-analysis identifies 11 new loci for anthropometric traits and provides insights into genetic architecture. Nat.Genet. 45, 501–512 (2013). 23563607 10.1038/ng.2606 PMC3973018

[b12] Liu C. T. *et al.* Genome-wide association of body fat distribution in African ancestry populations suggests new loci. PLoS.Genet. 9, e1003681 (2013). 23966867 10.1371/journal.pgen.1003681 PMC3744443

[b13] Shungin D. *et al.* New genetic loci link adipose and insulin biology to body fat distribution. Nature 518, 187–196 (2015). 25673412 10.1038/nature14132 PMC4338562

[b14] Deurenberg P., Deurenberg-Yap M. & Guricci S. Asians are different from Caucasians and from each other in their body mass index/body fat per cent relationship. Obes.Rev. 3, 141–146 (2002). 12164465 10.1046/j.1467-789x.2002.00065.x

[b15] Wen W. *et al.* Meta-analysis identifies common variants associated with body mass index in east Asians. Nat.Genet. 44, 307–311 (2012). 22344219 10.1038/ng.1087 PMC3288728

[b16] Wen W. *et al.* Meta-analysis of genome-wide association studies in East Asian-ancestry populations identifies four new loci for body mass index. Hum.Mol.Genet. 10.1093/hmg/ddu248 (2014). PMC4168820 24861553

[b17] Aschard H., Vilhjálmsson B. J., Joshi A. D., Price A. L. & Kraft P. Adjusting for heritable covariates can bias effect estimates in genome-wide association studies. Am. J. Hum. Genet. 96, 329–339 (2015). 25640676 10.1016/j.ajhg.2014.12.021 PMC4320269

[b18] Locke A. E. *et al.* Genetic studies of body mass index yield new insights for obesity biology. Nature 518, 197–206 (2015). 25673413 10.1038/nature14177 PMC4382211

[b19] Randall J. C. *et al.* Sex-stratified genome-wide association studies including 270,000 individuals show sexual dimorphism in genetic loci for anthropometric traits. PLoS.Genet. 9, e1003500 (2013). 23754948 10.1371/journal.pgen.1003500 PMC3674993

[b20] Pickrell J. K. *et al.* Understanding mechanisms underlying human gene expression variation with RNA sequencing. Nature 464, 768–772 (2010). 20220758 10.1038/nature08872 PMC3089435

[b21] Boyle A. P. *et al.* Annotation of functional variation in personal genomes using RegulomeDB. Genome Res 22, 1790–1797 (2012). 22955989 10.1101/gr.137323.112 PMC3431494

[b22] Lango A. H. *et al.* Hundreds of variants clustered in genomic loci and biological pathways affect human height. Nature 467, 832–838 (2010). 20881960 10.1038/nature09410 PMC2955183

[b23] Lecka-Czernik B., Lumpkin C. K. Jr. & Goldstein S. An overexpressed gene transcript in senescent and quiescent human fibroblasts encoding a novel protein in the epidermal growth factor-like repeat family stimulates DNA synthesis. MolCell Biol 15, 120–128 (1995). 10.1128/mcb.15.1.120 PMC231918 7799918

[b24] McLaughlin P. J. *et al.* Lack of fibulin-3 causes early aging and herniation, but not macular degeneration in mice. Hum.Mol.Genet. 16, 3059–3070 (2007). 17872905 10.1093/hmg/ddm264

[b25] Paternoster L. *et al.* Adult height variants affect birth length and growth rate in children. Hum.Mol.Genet. 20, 4069–4075 (2011). 21757498 10.1093/hmg/ddr309 PMC3177650

[b26] Dastani Z. *et al.* Novel loci for adiponectin levels and their influence on type 2 diabetes and metabolic traits: a multi-ethnic meta-analysis of 45,891 individuals. PLoS Genet. 8, e1002607 (2012). 22479202 10.1371/journal.pgen.1002607 PMC3315470

[b27] Kim Y. J. *et al.* Large-scale genome-wide association studies in East Asians identify new genetic loci influencing metabolic traits. Nat. Genet. 43, 990–995 (2011). 21909109 10.1038/ng.939

[b28] Kato N. *et al.* Meta-analysis of genome-wide association studies identifies common variants associated with blood pressure variation in east Asians. Nat.Genet. 43, 531–538 (2011). 21572416 10.1038/ng.834 PMC3158568

[b29] Takeuchi F. *et al.* Genome-wide association study of coronary artery disease in the Japanese. Eur. J. Hum. Genet. 3, 333–40 (2012). 21971053 10.1038/ejhg.2011.184 PMC3283177

[b30] Baik I., Cho N. H., Kim S. H., Han B. G. & Shin C. Genome-wide association studies identify genetic loci related to alcohol consumption in Korean men. Am. J. Clin. Nutr. 93, 809–816 (2011). 21270382 10.3945/ajcn.110.001776

[b31] Wells J. C. Sexual dimorphism of body composition. Best.Pract.Res.Clin.Endocrinol.Metab 21, 415–430 (2007). 17875489 10.1016/j.beem.2007.04.007

[b32] Cochran W. G. The Combination of Estimates from Different Experiments. Biometrics 10, 101–129 (1954).

[b33] Devlin B. & Roeder K. Genomic control for association studies. Biometrics 55, 997–1004 (1999). 11315092 10.1111/j.0006-341x.1999.00997.x

