# Supplementary material for: Genome-wide association studies in East Asians identify new loci for waist-hip ratio and waist circumference
Source: Sci Rep. 2016 Jan 20;6:17958. doi: 10.1038/srep17958 (PMC4726286; doi:10.1038/srep17958)
Supplement: Supplementary Information [file srep17958-s1.doc]

**Genome-wide association studies in East Asians identify new loci for waist-hip ratio and waist circumference**

Wanqing Wen, Norihiro Kato, Joo-Yeon Hwang, Xingyi Guo, Yasuharu Tabara, Huaixing Li, Rajkumar Dorajoo, Xiaobo Yang, Fuu-Jen Tsai, Shengxu Li, Ying Wu, Tangchun Wu, Soriul Kim, Xiuqing Guo, Jun Liang, Dmitry Shungin, Linda S. Adair, Koichi Akiyama, Matthew Allison, Qiuyin Cai, Li-Ching Chang, Chien-Hsiun Chen, Yuan-Tsong Chen, Yoon Shin Cho, Bo Youl Choi, Yutang Gao, Min Jin Go, Dongfeng Gu, Bok-Ghee Han, Meian He, James E. Hixson, Yanling Hu, Tao Huang, Masato Isono, Keum Ji Jung, Daehee Kang, Young Jin Kim, Yoshikuni Kita, Juyoung Lee, Nanette R. Lee, Jeannette Lee, Yiqin Wang, Jian-Jun Liu, Jirong Long, Sanghoon Moon, Yasuyuki Nakamura, Masahiro Nakatochi, Keizo Ohnaka, Dabeeru Rao, Jiajun Shi, Jae Woong Sull, Aihua Tan, Hirotsugu Ueshima, Chen Wu, Yong-Bing Xiang, Ken Yamamoto, Jie Yao, Xingwang Ye, Mitsuhiro Yokota, Xiaomin Zhang, Yan Zheng, Lu Qi, Jerome I. Rotter, Sun Ha Jee, Dongxin Lin, Karen L. Mohlke, Jiang He, Zengnan Mo, Jer-Yuarn Wu, E. Shyong Tai, Xu Lin, Tetsuro Miki, Bong-Jo Kim, Fumihiko Takeuchi, Wei Zheng, Xiao-Ou Shu

**SUPPLEMENTARY INFORMATION**

1. **SUPPLEMENTARY TABLES**
2. **DESCRIPTION OF PARTICIPATING STUDIES**
   1. **Stage I – Genome-Wide Association Meta-analysis**
   2. **Stage II –Replication Studies**
3. **SUPPLEMENTARY NOTES**
   1. **Quality Control (QC) Procedures**
   2. **Bioinformatics analysis**

**1. Supplementary Tables**

Supplementary Table 1. Description of studies participating in stage I.

Supplementary Table 2. Information on genotyping methods, quality controls for SNPs, imputation, and statistical analysis for studies participating in stage I.

Supplementary Table 3. Descriptive characteristics of studies participating in the initial meta-analysis of GWAS (stage I) and in the replication studies (stage II).

Supplementary Table 4. Associations in East Asian-ancestry populations of selected 33 SNPs in stage 1 and 2 with WC/WHR, with or without adjustment for BMI.

Supplementary Table 5. Associations in East Asian-ancestry populations of newly identified SNPs with WC/WHR, with or without adjustment for BMI, by stage or sex.

Supplementary Table 6. GIANT lookup associations for the newly identified SNPs.

Supplementary Table 7. Associations in East Asian-ancestry populations of newly identified SNPs with different traits.

Supplementary Table 8. Associations between SNPs at previously reported loci and WHRnoBMI in East Asian-ancestry populations.

Supplementary Table 9. Associations between SNPs at previously reported loci and WCnoBMI in East Asian-ancestry populations.

Supplementary Table 10. Associations between SNPs at previously reported loci and WHRadjBMI in East Asian-ancestry populations.

Supplementary Table 11. Associations between SNPs at previously reported loci and WCadjBMI in East Asian-ancestry populations.

Supplementary Table 12. Functional annotation of the newly identified SNPs and those SNPs in strong LD (r2>0.6).

Supplementary Table 13. The association of rs671 (G/A) and rs12229654 (T/G) with WC and WHR, with or without adjustment for BMI, in SGWAS, by alcohol consumption.

Supplementary Table 14. Associations between SNPs at previously reported loci and BMI in East Asian-ancestry populations.

**2. DESCRIPTION OF PARTICIPATING STUDIES**

**2.1 Stage I – Genome-Wide Association Meta-analysis**

**Shanghai Genome-Wide Association Studies (SGWAS)**

The Shanghai Genome-Wide Association Studies (SGWAS) include participants of the Shanghai Breast Cancer Study (SBCS), Shanghai Endometrial Cancer Study (SECS), Shanghai Breast Cancer Survival Study (SBCSS), Shanghai Women’s Health Study (SWHS), and Shanghai Men’s Health Study (SMHS). The SBCS and SECS are population-based, case-control studies, and the SBCSS, SWHS, and SMHS are ongoing population-based, prospective cohort studies. All participants of these studies were recruited in Shanghai during the same time period using similar study protocols. Structured questionnaires including the same core questions, were used to collected information on sociodemographic factors, reproductive history, lifestyle factors, and dietary habits. Anthropometrics, including weight, height, and waist and hip circumferences were taken by trained interviewers.

(1) The SBCS, described in detail elsewhere^1,2^, included two recruitment phases. During the initial phase (SBCS-I), 1,459 breast cancer patients and 1,556 controls were recruited between 1996 and 1998 through a rapid case-ascertainment system and the population-based Shanghai Cancer Registry. Blood samples were obtained from 1,193 (82%) cases and 1,310 (84%) controls. The second phase of participant recruitment (SBCS-II) was conducted between 2002 and 2005 using a protocol similar to the one used during the initial phase. A total of 1,989 incident cases and 1,918 community controls were recruited. The majority of cases (n = 1,932, 97.1%) and controls (n = 1,857, 96.8%) provided a blood sample or an exfoliated buccal cell sample. The age range of participants was 20-70 years with an average age of 50 years.

(2) The SECS, described in detail elsewhere^3^, included 1,204 endometrial cancer cases and 1,212 controls aged 30-69 years. The cases were newly diagnosed and identified through the population-based Shanghai Cancer Registry between 1997 and 2003. The controls were randomly selected from among the female residents of urban Shanghai through the Shanghai Resident Registry. Of the 1,204 cases and 1,212 controls, 857 cases and 837 controls donated a blood sample.

(3) The SBCSS, described in detail elsewhere^4^, is a population-based cohort study that recruited a total of 5,042 breast cancer cases diagnosed between April 1, 2002 and December 31, 2006 approximately six months after cancer diagnosis. In-person interviews were conducted to collect information on known breast cancer risk factors and anthropometrics by using a protocol and questionnaire similar to that used in the SBCS. Buccal cell samples were collected from 96% of study participants using a modified mouthwash method. Because of a time overlap during recruitment for the SBCS-II and the SBCSS, 1,469 breast cancer patients participated in both studies. DNA samples from SBCSS cases were scanned and contributed to the current study.

(4) The SWHS, described in detail elsewhere^5^, is a population-based cohort study of approximately 75,000 women who were aged 40-70 years at study enrollment and resided in seven geographically defined communities; 56,832 (75.8%) provided a blood sample. After approximately 10 years of follow-up to date, multiple disease outcomes, including breast cancer and diabetes, have been observed.

(5) The SMHS, described in detail elsewhere^6^, is a population-based cohort study of 61,504 Chinese men who were aged between 40 and 74 years, were free of cancer at enrollment, and lived in urban Shanghai, China. Recruitment for the SMHS was initiated in April 2002 and completed in June 2006. A total of 83,058 eligible male residents of eight communities in urban Shanghai were invited to participate by trained interviewers through in-person contact; 61,504 enrolled in the study with a response rate of 74.0%. Reasons for non-participation were refusal (21.1%), out of area during enrollment (3.1%), and other miscellaneous reasons including poor health or hearing problems (1.8%).

Participants included in the SGWAS were selected from the five studies described above. They constitute six case groups and a control group. The six case groups were: (i) the breast cancer group, which included 2,873 cases from the SBCS and SWHS; (ii) the endometrial cancer group, which included 840 cases from the SECS and SWHS ; (iii) the colorectal cancer group, which included 731 cases from the SWHS and SMHS, (iv) the pancreatic cancer group, which included 690 cases from the SWHS and SMHS, (v), the stomach and esophageal cancer group, which included 455 cases from the SWHS and SMHS, and (vi) the diabetes case group, which included 1,123 cases from the SWHS and SMHS. The control group included the remaining 3,001 participants, who served as the common control for the six disease groups. Thus, a total of 9,297 participants were included in the SGWAS, of whom 8,382 were women and 915 were men.

Anthropometric measurements taken at study recruitment for controls and for SWHS/SMHS participants were used in the analysis. For breast and endometrial cancer patients, self-reported weight at one year prior to diagnosis was used in the current analysis. Genomic DNA was isolated from peripheral blood for all participants except for 351 women from the SBCSS whose genomic DNA was extracted from buccal cells.

Genotyping methods and quality control (QC): Genomic DNA was extracted from buffy coats by using a Qiagen DNA purification kit (Valencia, CA) or Puregene DNA purification kit (Minneapolis, MN) according to the manufacturers’ instructions and then used for genotyping assays. The GWAS genotyping was performed at the Vanderbilt Microarray Shared Resource (VMSR) and Affymetrix Research Services Laboratory (ARSL) using the Affymetrix Genome-Wide Human SNP Array 6.0 (Affy6.0) platform, following Affymetrix’s protocols. In each of the 96-well plates for Affymetrix SNP 6.0 genotyping, three positive QC samples purchased from Coriell Cell Repositories (<http://ccr.coreill.org/>) were included. SNP data obtained from 227 positive quality control samples showed a very high concordance rate of called genotypes (mean, 99.85%; median, 100%). SNPs that showed genotyping call rates or concordance rates of less than 95% among the QC samples were excluded and the remaining samples were recalled by using Birdseed calling algorithm v2. In addition, a series of datasets were used to assess cross-genotyping platform validation. These included the following sets of SNPs that are on the Affymetrix SNP Array 6.0 and had been genotyped previously using various platforms for a subset of participants included in the GWAS scan: 1) 669 SNPs genotyped for 1,035 participants by using Affymetrix Target Genotyping System; 2) 17 SNPs genotyped for 1,091 participants by Taqman; and 3) 251 SNPs genotyped for 108 participants by using Sequenom. These three sets of SNPs served as cross-platform sample verification during the laboratory process. The mean concordance rates were 99.5%, 98.5%, and 98.9% for Affymetrix Targeted Genotyping, Taqman, and Sequenom, when compared with the Affymetrix SNP Array 6.0.

*Marker exclusion criteria:* The following quality control criteria were applied to assure the data quality of each SNP: 1) MAF <0.01; 2) call rate <95% in both VMSR and ARSL data; 2) bad genotyping cluster; 3) concordance rate <95% among duplicated QC samples in both VMSR and ARSL data; 4) concordance rate ≥95% among duplicated QC samples within VMSR and within ARSL data, but <95% in combined VMSR and ARSL data; 5) significant difference in allele frequency (*P*<0.01) between the breast cancer cases genotyped in the VMSR and ARSL data and the breast cancer controls genotyped in VMSR and ARSL, and the differences were in the same direction in breast cancer cases and controls; 6) SNPs with a call rate <95% or QC concordance rate <95% or bad genotyping cluster within VMSR data were set to missing in the VMSR dataset. These criteria were also used for the ARSL dataset. After applying the QC filter, 690,947 SNPs remained for the analyses and were used for imputation.

*Individual exclusion criteria:* The gender of all study participants was confirmed according to the X chromosome genotyping data. Multidimensional scaling (MDS) analyses based on pairwise IBS showed that all participants in the present study were clustered closely with HapMap Asians. We also excluded samples that had: 1) call rate <95%; 2) contaminated samples (based on inbreeding coefficients), samples with mixed-up labels, or duplicated samples based on IBD estimate; 3) first-degree relatives, such as parent-offspring and full siblings based on IBD estimate. A total of 21 participants were excluded. Other relationships (21 half-sibling/aunt-niece pairs and 26 first-cousins/grandchild-grandparent pairs) remained in the dataset.

*Imputation methods.* Genotypes were imputed using the program MACH (http://www.sph.umich.edu/csg/abecasis/MACH/download/), which determines the probable distribution of missing genotypes conditional on a set of known haplotypes, while simultaneously estimating the fine-scale recombination map. Phased autosome SNP data from HapMap Phase II Asians (release 22) were used as the reference. To test for associations between the imputed SNP data with BMI, linear regression (additive model) was used, in which SNPs were represented by the expected allele count, an approach that takes into account the degree of uncertainty of genotype imputation (<http://www.sph.umich.edu/csg/abecasis/MACH/download/>).

**Taiwan Genome Wide Association Study (TGWAS):**

This study included 1,000 random samples from the Han-Chinese Cell and Genome Bank in Taiwan and 999 participants from the Taiwan type 2 diabetes study^7^. The Han-Chinese Cell and Genome Bank in Taiwan^8^ includes more than 3,300 healthy controls who were recruited via a stratified, 3-stage probability clustering sampling scheme through a registry of all the 329 non-aboriginal townships or city districts in Taiwan. The study was approved by the institutional review board of Academia Sinica, Taiwan and written, informed consent was obtained from all participants. The Taiwan type 2 diabetes study included a total of 2,798 unrelated individuals (1,440 men and 1,358 women, participation rate ~60%) with type 2 diabetes, age >20 years^7^. All of the type 2 diabetes cases were diagnosed according to medical records and fasting plasma glucose levels by using American Diabetic Association criteria. The study was approved by the institutional review board and the ethics committee of each institution. Written, informed consent was obtained from each participant in accordance with institutional requirements and Declaration of Helsinki Principles. For this meta-analysis, 999 participants recruited by the China Medical University Hospital, Taichung, Taiwan, were analyzed.

Genotyping and QC: Genomic DNA was extracted from peripheral blood using the Puregene DNA isolation kit (Gentra Systems, Minneapolis, MN, USA). Whole genome genotyping using the Illumina HumanHap550-Duo BeadChip was performed by deCODE Genetics (Reykjavík, Iceland). Genotype calling was performed using the standard procedure implemented in BeadStudio (Illumina, Inc., San Diego, CA, USA), with the default parameters suggested by the platform manufacturer. QC of genotype data was performed as described in detail elsewhere^7,9^. In brief, for each sample genotyped in this study, the average call rate was 99.92±0.12%. After applying stringent QC criteria, high-quality genotypes for 516,737 SNPs (92.24%) were obtained, with an average call rate of 99.92±0.24%. SNPs were excluded if they: 1) were nonpolymorphic among both cases and controls, 2) had a total call rate <95% among cases and controls combined, 3) had a MAF <5% and a total call rate <99% among cases and controls combined, or 4) had significant deviation from HWE among the controls (*P*<10^−7^).

**Cardiometabolic Risk in Chinese (CRC) Study:**

The Cardiometabolic Risk in Chinese (CRC) Study is a community-based health examination survey of 6,431 individuals (aged 18-93 years; 53.7% men) who were randomly selected from residents living in the urban area of Xuzhou, China in 2009. Written consent was obtained from all participants. The study was reviewed and approved by the ethics committee of the Xuzhou Central Hospital, Jiangsu, China. A total of 811 study samples were included in a GWAS that was carried out on Illumina Human660-Quad BeadChips at the Chinese National Human Genome Center in Shanghai, China. Genotype clustering was conducted with Illumina BeadStudio 3.3 software. Height was measured to the nearest 0.5 cm without shoes and body weight was measured to the nearest 100 grams without shoes. Waist circumference, an index of total abdominal fat, was measured at the mid-point between the lowest rib margin and the iliac crest. Hip circumference was measured at the maximum protuberance of the buttocks. We calculated waist to hip ratio (WHR) as waist circumference (cm) divided by hip circumference (cm). BMI was calculated as weight (in kilograms) divided by height (in meters) squared.

**Fangchenggang Area Male Health and Examination Survey (FAMHES) Study**

Design, Population Recruitment, and Sample Size: The FAMHES is a population-based study conducted among non-institutionalized Chinese men aged 17 to 88 years in Guangxi and was designed to investigate the effects of environmental and genetic factors and their interaction with the development of age-related chronic disease. A comprehensive demographic and health survey was conducted among 4,303 continuous men who participated in a large-scale physical examination in Fangchenggang First People’s Hospital Medical Centre from September 2009 to December 2009. All participants provided written informed consent and the study received local ethics committee approval. The current study was confined to men aged 20 to 69 years. All participants reported themselves to be of Chinese southern Han origin and to be free of diabetes mellitus, coronary heart disease, stroke, hyperthyroidism, rheumatoid arthritis, cancer, and impaired hepatic or renal function. For the association study, 2,018 men with anthropometric measurements were included.

Source of anthropometric information: Anthropometric measurements were performed by trained personnel using a standardized protocol. Body weight and height were measured without shoes to the nearest 0.1 kg and 0.1 cm, respectively. Body mass index (BMI) was then calculated as weight (kg)/height (m^2^). Waist circumference was measured midway between the lowest rib and the iliac crest to the nearest 0.1 cm, and hip circumference was taken over the widest part of the gluteal region. The waist to hip ratio (WHR) was then calculated as waist circumference (cm)/hip circumference (cm).

Genotyping platform: The study populations were genotyped using the Illumina HumanOmni1-Quad BeadChip (Illumina, CA, USA). All of these experiments were performed at the same institute by the same technical staff. 19 individuals were excluded for being a suspected first-degree relative of an included individual based on genome-wide genotyping data. Individuals with a call rate <95% were excluded from analysis. SNPs with HWE *P*< 10^-4^ or a call rate <95% or a MAF <1% were excluded from analysis.

**Nutrition and Health of Aging Population in China (NHAPC) Study**

Study population: A population-based sample consisting of 2,888 unrelated individuals (1,250 men and 1,638 women) aged 50–70 years was obtained from the Nutrition and Health of Aging Population in China study, which aimed to examine environmental and genetic factors in relation to metabolic diseases. Details of the study design and inclusion/exclusion criteria have been described elsewhere^10^. Data on demographic variables, health status, health behavior, and physical activity were collected using a standardized questionnaire. All participants were required to fast overnight (≥7 hours) before a physical examination and blood collection. Body weight and height, waist and hip circumference, and blood pressure were measured by trained staff and physicians using standard protocols. Height and weight were measured with participants dressed in light-weight clothing without shoes, and BMI was calculated as weight (kg) divided by height squared (m^2^). Waist circumference was measured at the mid-point between the lowest rib and the iliac crest, after inhalation and exhalation. Hip circumference was measured at the maximum protuberance of the buttocks, and WHR was calculated as waist circumference (cm) divided by hip circumference (cm). The study was approved by the Institutional Review Board of the Institute for Nutritional Sciences and written informed consent was obtained from all participants.

SNP genotyping and quality control: Genomic DNA was extracted from peripheral blood leukocytes by the salting-out procedure (available at <http://humgen.wustl.edu/hdk_lab_ manual/dna/dna2.html>). All samples were genotyped using the Illumina Human660W-Quad BeadChip at Bio-X Center and Chinese National Human Genome Center in Shanghai. The data generated by the chip were loaded into separated GenomeStudio files according to the genotyping locale, and genotype calling was carried out by clustering with a no-call threshold of 0.15 on the GenCall score. Genotypes were merged into one file for quality control. Samples were excluded for genotype call rates < 97%, excessive heterozygosity, gender mismatches between the reported and genetically inferred gender, or duplicates among other samples. Principal component analysis was used to assess the population structure of the samples and detected outliers along the first ten eigenvectors, which were excluded from further analyses. SNPs with genotype a call rate < 99%, a MAF < 1%, or deviation from Hardy-Weinberg equilibrium (*P* <10^-3^) were also excluded. A total of 468,786 SNPs remained for genome-wide imputation and association analyses.

Genome-wide imputation and association analysis: Genome-wide imputation was performed on all samples that passed quality control procedures based on 468,786 autosomal SNPs with a MAF ≥ 1% by using the program IMPUTE (version 2.1.2) using 180 phased CHB+JPT haplotypes from HapMap Phase 2 release 22 as the main reference. We also included 170 phased CHB+JPT haplotypes from HapMap Phase 3 release 2 as another reference to increase imputation accuracy by the program IMPUTE. Overlapping samples and mismatched strands of SNPs between these two data sets had been corrected before imputation. All imputed SNPs with an estimated call rate < 99%, MAF < 1%, deviation from Hardy-Weinberg equilibrium (*P* < 10^-6^), or proper-info value of imputed SNPs ≤ 0.5 were excluded. We checked all pairs of samples showing pairwise IBD > 0.375 computed using the program PLINK (version 1.07), and one sample in each pair was carefully removed to avoid first-degree cryptic relationships. Finally, 2,888 samples and more than 2.3 million SNPs remained for the genome-wide association analyses. A linear regression model was used to regress quantitative phenotypes and a logistic regression model was used to regress dichotomous phenotypes by the program SNPTEST (version 2.2.0). All regression analyses were carried out using an additive genetic model and represented each SNP by the expected number of copies of the coded allele.

**The Genetic Epidemiology Network of Salt Sensitivity (GenSalt)**

GenSalt study participants were recruited from six sites in rural areas of northern China from October 2003 to July 2005^11^. The selection of these study sites was based on the homogeneity of the study population with regard to ethnicity and environmental exposures, including lifestyle, nutritional factors, and habitual dietary intake. The residents in these regions are of the Han ethnicity, the ethnic majority in China. A community-based blood pressure screening was conducted among persons aged 18-60 years in the study villages to identify potential probands and their families for the study. Those with a mean systolic blood pressure between 130-160 mmHg and/or diastolic blood pressure between 85-100 mmHg and no use of antihypertensive medications and their spouses, siblings, and offspring were recruited as volunteers for a dietary intervention study. In general, individuals who had stage-2 hypertension, secondary hypertension, use of antihypertensive medications, history of clinical cardiovascular disease, diabetes, chronic kidney disease, pregnancy, or heavy alcohol use were excluded from the study. A total of 1,906 individuals (1,010 men and 896 women) met the eligibility criteria for the dietary intervention study. Of these individuals, 1,843 (96.7%) completed the entire 21-day dietary intervention and were included in the GWAS. The completeness of the study questionnaire data, blood pressure and anthropometric data, and blood and urine sample collection is near 100%. The institutional review boards at all participating institutes approved the study, and written, informed consent was obtained from each participant.

A standard questionnaire was administered by trained staff at the baseline examination to collect information on demographic characteristics, personal and family medical history, and lifestyle risk factors (including cigarette smoking, alcohol consumption, and physical activity). Three blood pressure measurements were obtained each morning during the 3-day baseline examination by trained and certified observers using a random–zero sphygmomanometer according to a standard protocol. Blood pressure was measured with the participant in a seated position after 5 minutes of rest. In addition, participants were advised to avoid consumption of alcohol, coffee or tea, or cigarettes and exercise for at least 30 minutes prior to their blood pressure measurements. Body weight, height, and waist circumference were measured twice with the participant in light indoor clothing without shoes. Waist circumference was measured one cm above the participant’s navel during minimal respiration. Overnight (≥8 hours) fasting blood specimens were obtained for measurement of glucose and lipids. Plasma glucose was measured using a modified hexokinase enzymatic method (Hitachi automatic clinical analyser, model 7060, Japan). Concentrations of total cholesterol, HDL-cholesterol, and triglycerides were assessed enzymatically using commercially available reagents. Concentration of LDL-cholesterol was calculated by means of the Friedewald equation for participants who had less than 400 mg/dL triglycerides: LDL cholesterol=total cholesterol–HDL cholesterol–triglycerides/5.

Lymphocytic DNA samples were obtained from GenSalt family members (probands, parents, spouses, siblings, and offspring). Genome-wide SNPs were genotyped using Affymetrix® Genome-Wide Human Array 6.0 at the Affymetrix genotyping facility. After removing sex-linked SNPs, mitochondrial SNPs, and ‘unassigned’ SNPs that had no annotated chromosomal location, 871,166 SNPs were chosen for examination. Strict procedures for extensive QC were used to check the data for any obvious errors, remove uninformative data, and find and remove all Mendelian errors in three stages. In stage 1, we removed participants with gender discrepancies between reported sex and that estimated by PLINK ^12^and those who had potential pedigree errors, as determined by GRR^13^. In stage 2, we removed monomorphic SNPs, Affymetrix ‘housekeeping’ SNPs, SNPs with missing rates of >25% or a MAF of <1%. In the final stage, Mendelian errors were found and removed using PLINK^12^ and PedCheck^14^. After the QC process, 820,015 autosomal SNPs from 1,881 participants remained. An additional 1,792,556 SNPs were imputed from a HapMap reference panel using data on 90 individuals from the JPT and CHB populations. The QC processes removed imputed SNPs with R^2^<0.3, MAF<1%, Hardy-Weinberg *P*-value <10^-6^, or Mendelian errors. Finally, 2,216,774 autosomal SNPs were used for GWAS analyses.

To estimate the association between BMI and SNPs, a mixed linear model was used for association test, and family structure was taken into account by treating it as a random effect.

**Dongfeng-Tongji Cohort Study (DFTJ)**

Study participants. In the present study, the GWAS stage included 1,461 unrelated healthy Chinese from the Dongfeng-Tongji (DFTJ) cohort study, which has been described in detail elsewhere^15^. Briefly, the DFTJ cohort, which included 27,009 retired employees from a state-owned automobile enterprise in China, was designed to investigate the effect of environmental and genetic factors and their interaction with chronic diseases such as cardiovascular disease and cancer. All participants included in the DFTJ cohort were recruited at health check-ups; 1,452 participants who met stringent quality control criteria were included in the GWAS. Standing height and body weight were measured with participants with light indoor clothing and without shoes. BMI was calculated as the individual's body mass (kg) divided by the square of his (her) height (m^2^). Waist circumference was measured midway between the lowest rib and the iliac crest to the nearest 0.1 cm. All participants provided informed consent and the ethical committees in the Tongji Medical College approved the research project.

Genotyping methods and quality control. Genotyping for the GWAS was done using Affymetrix Genome-Wide Human SNP Array 6.0 chips. In total, we genotyped 906,703 SNPs for the GWAS. After stringent quality control procedures [minor allele frequency (MAF) ≥ 0.01, Hardy-Weinberg Equilibrium (HWE) ≥ 0.0001, SNPs call rate ≥ 95%, and individuals with a genotyping call rate ≥ 95% ], the genotypes of 1,452 participants with 658,288 autosomal SNPs were used in subsequent analyses with an overall call rate of 99.68%. We used MACH 1.0 software to impute untyped SNPs using the LD information from the HapMap phase II database (CHB+JPT as a reference set (2007-08_rel22, released 2007-03-02). SNPs with a MAF >1% and with MACH RSQR >0.3 were kept for the association analyses. A total of 2,241,204 genotyped and imputed SNPs remained for further association analyses. We used ProbABEL software to conduct the association studies with imputation data^16^.

**Singapore Prospective Study Program (SP2)**

Population: The Singapore Prospective Study Program (SP2) is a cross-sectional study of 6,968 adult Singaporean Chinese, Malay and Asian-Indian men and women, aged 24-95 years.  Individuals who participated in previous cross sectional studies, the Thyroid and Heart Study 1982–1984^17^, National Health Survey 1992^18^, National University of Singapore Heart Study 1993–1995^19^, or National Health Survey 1998^20^, were invited to participate. All studies involved a random sample of individuals from the Singapore population, aged 24 to 95 years, with disproportionate sampling stratified by ethnicity to increase the number of minority ethnic groups (Malays and Asian Indians). Individuals who were successfully re-contacted and gave informed consent answered a questionnaire and attended a clinic examination. Height (m) and weight (kg) were measured similarly in all datasets using standard protocols and were used to derive BMI as weight over height-squared (kg/m^2^). Institutional review board approval was provided by the National Healthcare Group domain specific review board.

Genotyping methods and quality control: Genotyping assays were conducted together as these studies were originally part of a Singaporean Chinese case-control study of type 2 diabetes. A total of 3,066 Chinese adults from the SP2 were genotyped using 1Mduov3 (N=1,016), HumanHap 610Quad (N=1,467), and Hap550 (N=583).

The average SNP concordance rate between chips for the post-quality control (QC) duplicated samples was computed based on 531,805 post-QC common SNPs between 1Mduov3 and 610 Quad chips and 496,653 post-QC common SNPs between the 1Mduov3 and 550 chips. The mean concordance was >95%, and 5 discrepant SNPs were removed (rs10953303, rs11075260, rs1447826, rs274646, and rs430794). For each array in each cohort, a first round of clustering was performed with the proprietary clustering files from Illumina (GenCall). Samples achieving a 99% call rate were subsequently used to generate local clusterfiles (GenTrain), which were used for a final round of genotype calling. A threshold of 0.15 was implemented on the GenCall score to decide on the confidence of the assigned genotypes.

Samples were removed based on the following conditions: sample call rates of less than 95%, excessive heterozygosity, cryptic relatedness, discordant ethnic membership, or gender discrepancy. Bivariate plots of sample call rates and heterozygosity, defined as the proportion of heterozygous calls of all valid autosomal genotypes in an individual, were used to assess the overall distribution of missingness and heterozygosity across all the samples. Identity-by-state measures were performed by pair-wise comparison of samples to detect cryptic relatedness such as monozygotic twins, full-sibling pairs, and parent-offspring pairs. One sample from each relationship was excluded from further analysis and where duplicate samples had been genotyped in different SNP-arrays, samples from the denser array was retained. Population structure ascertainment to prevent confounding of study results was performed by using principal component analysis (PCA) with 4 panels from the International HapMap Project ([http://hapmap.ncbi.nlm.nih.gov](http://hapmap.ncbi.nlm.nih.gov/)) and the Singapore Genome Variation Project (<http://www.nus-cme.org.sg/SGVP>) with a thinned set of SNPs to reduce linkage disequilibrium (LD). Individuals who showed ethnic membership discordant from their self-reported ethnicity were excluded from the analysis. A total of 2,431 samples with BMI, WHR, and waist circumference data were available after sample QC procedures.

We excluded sex and mitochondrial SNPs, together with gross Hardy-Weinberg equilibrium (HWE) outliers (*P*-value <1x10^-4^). SNPs that were monomorphic or rare (minor allele frequency (MAF) <1%) and SNPs with low call-rates (<95%) were also excluded. Where more than one chip was used for genotyping, Mantel-extension tests were carried out to detect differences in allele frequencies of SNPs between the chips; 62 such SNPs were detected and removed from the analyses.

Imputation methods: Imputation procedures were performed using IMPUTE v0.5.0.^21^ and genotype calls were based on HapMap Phase 1 and 2 East-Asian samples (CHB and JPT) of NCBI build 36 for all Chinese samples^22^. Actual genotyped calls were placed back into the files and only imputed SNPs, and a posterior probability ≥0.90 and call-rate ≥95% were used. A total of 1,745,788 SNPs were available for subsequent analyses after imputation and QC procedures.

**The Multi-Ethnic Study of Atherosclerosis (MESA)**

The Multi-Ethnic Study of Atherosclerosis (MESA) is a study of the characteristics of subclinical cardiovascular disease (disease detected non-invasively before it has produced clinical signs and symptoms) and the risk factors that predict progression to clinically overt cardiovascular disease or progression of subclinical disease. MESA researchers study a diverse, population-based sample of 6,814 asymptomatic men and women aged 45-84 years. Thirty-eight percent of the recruited participants are white, 28 percent African-American, 22 percent Hispanic, and 12 percent Asian^23^. Of them, 775 Chinese with genotyping data participated in the current analysis. The participants were recruited from six field centers across the United States. Written informed consent was obtained from each participant. Height and weight were measured to the nearest 0.1 cm and 0.5 kg, respectively. Body mass index (BMI) was defined as weight in kilograms divided by height in meters squared and was used as a measure of overall obesity. Girths (waist at the umbilicus and hips at the maximal circumference of buttocks) were measured to the nearest 0.1 cm using a steel measuring tape (standard 4 oz. tension). The waist/hip ratio (WHR) and waist circumference were used as indices of body fat distribution. All measurements used in this paper were obtained at the first MESA study visit.

Genotyping was performed by using the Affymetrix Genome-Wide Human SNP Array 6.0. IMPUTE version 2.1.0 was used to perform imputation for the MESA Chinese participants (chromosomes 1-22) using HapMap Phase I and II - CEU+YRI+CHB+JPT (rel#22, BCBI Build 36, dbSNP b126), which was used as the reference panel. SNPs with genotyping call rates <0.95, MAF <0.02, HWE *P*-value <0.000001, or Rsq <0.3 were removed from the analysis. Association tests were performed by SNPTEST v2^21^.

**Korea Genome Wide Association Studies**

The Korea Association REsource (KARE) project has been described previously^24^. This project was initiated in 2007 to undertake a large-scale GWAS. A total of 10,038 participants aged between 40 and 69 years were recruited through two population-based prospective cohort studies conducted in the Ansung (n=5,018) and Ansan (n=5,020) areas of South Korea. Both cohorts were designed to allow longitudinal prospective studies and adopted the same investigational strategy. More than 260 traits have been extensively examined through epidemiological surveys, physical examinations, and laboratory tests. Included in the current analysis were 8,838 participants with anthropometric measurements and genomic genotyping data.

Health Examinee (HEXA) shared control study. The HEXA cohort is one of the Korean Genome and Epidemiology Study (KoGES) population-based cohorts which were initiated in 2001 aiming to identify risk factors of life-style related complex diseases such as type 2 diabetes, hypertension, and dyslipidemia. Approximately 3,700 of 1,200,000 individuals aged 40-69 years from the HEXA cohort were randomly selected as a shared control group for the Korean cancer and coronary artery disease (CAD) GWAS^25^. Genotyping was conducted with the Affymetrix Genome-Wide Human SNP array 6.0 in 2008.

Health2 cohort. Samples were selected from another community-based cohort provided by the Health2 study. We examined 1,816 individuals selected from the 8,500 participants. The participants were aged 40- 69 years. The study objective and the strategy for clinical measurements of the Health2 cohort were similar to those of the discovery stage participants^24,25^.

Cardiovascular disease association study (CAVAS) cohort. Study participants were selected from an ongoing population-based cohort, the Korean Genome and Epidemiology Study (KoGES). Participants were recruited from among residents aged 40-69 years of three rural cities-Yangpyeong in Gyeonggi-do province, Goryeong in Gyeongsangbuk-do province, and Namwon in Jeollabuk-do province, Korea. A total of 8,702 men and women were recruited from 2004 through 2008 for the baseline study. Of them, 4,052 healthy participants with no history of hypertension, type 2 diabetes, hyperlipidemia, heart disease, blood vessel disease of the brain, or cancer were selected for SNP genotyping.

Genotyping methods and quality control: A total of 19,891 Korean adults were genotyped using the Affymetrix Genome-Wide Human SNP array 5.0 (KARE: n=9,603), Affymetrix Genome-Wide Human SNP array 6.0 (HEXA: n=4,302 and Health2: n=1,952) and Illumina HumanOmniI Quald vI (Rural: n=4,034). Genotypes were called using the Bayesian Robust Linear Modeling using the Mahalanobis Distance (BRLMM) for KARE, the Birdseed for HEXA and Health2, the BeadStudio for Rural, respectively. Samples that exhibited the following properties were excluded: low genotyping calls (< 96% for Affymetrix and < 98% for Illumina), excessive heterozygosity, sex inconsistency, discordant ethnic membership, or cryptic relatedness. Markers with high missing gene call rates (>5%), low MAF (<0.01), or significant deviation from Hardy-Weinberg equilibrium (*P*< 1 ×10^-6^) were excluded.

Imputation methods. Imputation analysis was performed using IMPUTE against all of the HapMap Asian (JPT+CHB) population (release 22/NCBI, build 36, and dbSNP build 126) as a reference panel. We used posterior probability to call the genotype from imputation data and then performed association analyses on imputed data. Of these, SNPs in each cohort with a posterior probability score < 0.90, high genotype information content (info < 0.5), HWE (*P* < 1 × 10^-7^), and MAF < 0.01 were dropped.

**The Korean Cancer Prevention Study-II (KCPS-II)**

The KCPS-II has been described previously^26^. The KCPS-II included 266,258 individuals, aged 20-77 years, who visited 16 health promotion centers across South Korea from April 2004 to December 2008. Participants were interviewed at baseline to obtain exposure data. Cancer diagnoses were identified through 2008 using data from the national cancer registry and hospitalization records. Mortality outcomes were ascertained through 2009 by reviewing death certificates. A computerized search of death-certificate data from the National Statistical Office in Korea was performed using the unique identification number assigned at birth. For the study, we selected 325 CRC patients who provided a blood sample. Cancer-free cohort members (N= 977) were randomly selected as controls. Therefore, a total of 1,302 individuals were genotyped.

Genotyping methods and quality control: Cohort samples were genotyped on the Affymetrix Genome-wide Human SNP Array 5.0 at DNALink. For the data obtained from this chip, the following internal quality control (QC) measures were used: the QC call rate (dynamic model algorithm) always exceeded 86%, and the heterozygosity of X chromosome markers was used to identify the gender of each individual. Genotype calling was performed with the Birdseed (v2) algorithm. A total of 1,004 individuals were genotyped via this platform in the first discovery phase. However, 10 of 1,004 individuals were removed because of low genotyping call rates (<95%). PLINK (v1.07) ^12^was used to estimate identity by state (IBS) over all SNPs, and four individuals were shown to be biological relatives, so one member of each pair was excluded. Eleven individuals were also excluded as a result of gender mismatches. Therefore, 979 individuals were available for this genome-wide analysis. A default set of 400,794 SNPs were used for further analysis, as recommended by Affymetrix. For quality assurance screening, we flagged SNPs with genotype call rates < 95%, minor allele frequencies (MAF) < 0.01, and SNPs showing deviation from Hardy-Weinberg equilibrium (HWE) at *P* < 0.0001. The final set of acceptable markers included 317,859 autosomal SNPs.

**Cardio-metabolic Genome Epidemiology (CAGE)**

The Cardio-metabolic Genome Epidemiology (CAGE) Network is an ongoing collaborative effort to investigate genetic and environmental factors and their interactions affecting cardiometabolic traits/disorders among Asians, including the Japanese^27,28^. CAGE participants were recruited in a population-based or hospital-based setting, depending on the design of the member studies. Participation rates varied among the member studies (approximately from 25% in the community-based survey to 80% in the work place-based survey). From this network, a total of 415 Japanese samples (265 men and 150 women; age range 38-95 years, mean 69 years) were used for a genome-wide association study (GWAS) of measures of abdominal obesity. Participants’ height, body weight and waist circumference were measured by trained personnel using standard anthropometric techniques. In the CAGE Network, all participants provided written informed consent, and studies were approved by local Research Ethics Committees and/or Institutional Review boards.

Genotyping methods and quality control: Genotyping was performed with Infinium HumanHap550/Human610-Quad BeadArray (Illumina, San Diego, CA, USA), which interrogated 550K/610K SNPs, according to the manufacturer’s protocol. This set of SNP markers reportedly captures 87% of common SNPs with an LD coefficient of *r^2^* > 0.8 in the HapMap JPT and CHB populations (according to the manufacturer’s brochure). Assay accuracy and reproducibility were measured by using DNA from CEU samples genotyped as part of the HapMap project [http://www.hapmap.org]. Genotype calling was performed using BeadStudio software (Illumina), and genotype calls with a ‘GenCall’ Score <0.53 were dropped from the analysis. The GenCall Score measures the reliability of genotype calls based on the clustering of dye intensities (www.illumina.com/downloads/ GenCallTechSpotlight.pdf).

QC of SNPs and samples was performed as previously described^28^. Briefly, data cleaning and analysis were performed using PLINK software (version 1.06)^12^. Among the assayed SNPs, we excluded SNPs for the following reasons: 1) genotype call rate <0.95; 2) significant (*P*<10^-6^) deviation from HWE; or 3) MAF <0.01. The remaining 451,382 SNPs were analyzed in the genome scan. The average call rate for the 451,382 QC’d SNPs was 99.7% in 2,194 samples tested for an association with obesity.

**The Cebu Longitudinal Health and Nutrition Survey (CLHNS)**

The Cebu Longitudinal Health and Nutrition Survey (CLHNS) is an ongoing community-based birth cohort study that began in 1983^29^. The baseline survey randomly recruited 3,327 pregnant women from the Metropolitan Cebu area, the Philippines in 1983-84 (3,080 singleton live births), and since followed them and their offspring to the present. Trained field staff conducted in-home interviews and collected anthropometric measurements at each visit. Blood samples for biomarker measurement and DNA extraction were obtained in 2005. For this study of 1,779 CLHNS mothers, weight, height, and the calculated BMI were ascertained in the 2005 survey.

Genotyping methods and quality control: SNP genotyping, quality control and genotype imputation have been previously described ^30^. Briefly, SNP genotyping was performed with the Affymetrix Genome-Wide Human SNP Array 5.0, using the standard protocol recommended by the manufacturer. Genotype calling was performed using Birdseed (version 2). The sample call rate was 99.6%. For marker quality control, SNPs with poor mapping, call rate < 90%, and/or deviation from Hardy-Weinberg equilibrium (*P*<10^-6^) were removed prior to imputation. We applied a hidden Markov model algorithm implemented in MACH to impute genotypes in CLHNS mother samples based on HapMap (Release 22) CHB+JPT samples. A total of 2,206,824 SNPs with MAF ≥ 0.01 and imputation quality Rsq > 0.3 were tested for associations using the software mach2qtl.

- 1. **Stage II – Replication Studies**

**Vanderbilt University site**

The *de novo* genotyping conducted at Vanderbilt University included 4,905 Chinese women and 1,438 Chinese men, a total of 6,343 individuals, who were subjects from the SBCS, SWHS, SMHS, and SECS. These studies are described above in the description of the SGWAS. Anthropometrics, including weight, height, and waist and hip circumferences were taken by trained interviewers in all those parent studies. The individuals selected for *de novo* genotyping were independent of the individuals included in the SGWAS. The genotyping was performed using the Sequenom iPLEX MassARRAY® at Vanderbilt University.

**CAGE-Fukuoka/KING Study**

Participants in two study panels (Fukuoka and KING Study panels of the CAGE network) participated in the stage II analysis.

Fukuoka Study panel: This study includes participants in the baseline survey of the Kyushu University Fukuoka Cohort Study, which was designed to investigate lifestyle factors and genetic susceptibility to so-called lifestyle-related diseases such as cardiovascular disease, cancer, and diabetes mellitus. Eligible individuals were residents of the East Ward of Fukuoka City aged 50-74 years. Some areas in the Ward were excluded because of potential emigration, sparse population, and remote distance. Of the 53,927 individuals who were contacted by mail, a total of 12,959 participated in the baseline survey conducted from February 2004 to August 2007. A brief description of the methods used in the baseline survey is available elsewhere^31^. After excluding 8 individuals who withdrew from the study, 1 individual for duplicate participation, and 1 individual for mental incompetence, the cohort comprised 12,949 participants. Of them, 12,629 gave informed consent for genetic analysis, and a total of 12,569 participants completed the questionnaire and also provided DNA for genotyping of SNPs. Participants’ height, body weight, and waist and hip circumference were measured by trained personnel using standard anthropometric techniques.

KING Study panel: The Kita-Nagoya Genomic Epidemiology (KING) study (ClinicalTrials.gov identifier: NCT00262691) is an ongoing community-based prospective observational study of the genetic basis of cardiovascular disease and its risk factors. The study recruited 3,975 Japanese subjects aged 50-80 years, who underwent community-based annual health checkups between May 2005 and December 2007, as described in detail previously^32^. Participants’ height, body weight and waist circumference (WC) were measured by trained personnel using standard anthropometric techniques.

Genotyping methods and quality control: Samples were genotyped using the Sequenom iPLEX MassARRAY® and/or the TaqMan assay (Applied Biosystems by Life Technologies, Carlsbad, CA) for SNPs identified in stage I. The genotype distribution of all tested SNPs was in Hardy–Weinberg equilibrium (*P*>10^−3^). We obtained successful genotyping call rates of >99% for all SNPs and >99% for all included samples.

A total of 12,569 participants from Fukuoka Study panel and 2,590 participants from KING Study panel who provided WHR and/or WC and genotyping data participated in the stage II analysis.

**The Japanese Millennium Genome Project (JMGP)**

JMPG is a consortium for studies of cardiovascular disease genetics, and a part of the JMGP consortium participated in this study. The Shigaraki study of Shiga University of Medical Science is a population-based longitudinal study based on Shigaraki-city residents of which participants were recruited through an annual community-based medical check-up process. The Anti-Aging Center Cohort study conducted by Ehime University is a longitudinal study based on consecutive participants in the medical check-up program at Ehime University Hospital Anti-aging Center. This medical check-up program was specifically designed for the general population to evaluate age-related disorders, including arteriosclerosis, cardiovascular diseases, physical function, and cognitive impairment.

In the both cohorts, anthropometric parameters were obtained from personal health records evaluated at the check-ups. In brief, body height and body weight were measured to the first decimal place using a digital scale. Waist circumference was measured at the umbilicus level by using a measuring tape after a deep breath.

Genotyping methods and quality control: Samples were genotyped using the TaqMan assay (Applied Biosystems by Life Technologies, Carlsbad, CA). We obtained successful genotyping call rates of 96.9 to 99.5% for all included samples.

**3. SUPPLEMENTARY NOTES**

**3.1. Quality Control (QC) Procedures**

The following QC procedures were recommended for each participating study. SNPs and/or individuals were excluded either in the primary analysis conducted by each participating study or at the meta-analysis stage (Supplementary Table 2) for the following reasons: (1) individuals or SNPs with a call rate < 90%; (2) SNPs with *P*<1.0×10^-6^ for the Hardy-Weinberg violation test; (3) SNPs with a MAF<1%; (4) imputed SNPs with low imputation quality (r-hat <0.3 for MACH or proper-info <0.5 for IMPUTE); (5) individuals with first-degree cryptic relationships as shown via an identity-by-descent (IBD) analysis; (6) and samples that were potentially contaminated.

Supplementary Table 2 summarizes the specific QC procedure adopted by each study.

**3.2. Bioinformatics analysis**

We annotated those newly identified SNPs candidate causative variants for potential functional significance based on epigenomic data from the ENCODE project. A publically available tool HaploReg2 were used to identify all SNPs with significant levels of LD > 0.6 for each newly identified SNP and their functional annotation were extracted to examine whether they lie in enhancer or promoter regions ^33^. We also annotated those SNPs using chromHMM annotation across nine ENCODE cell lines: HMEC, GM12878, H1-hESC, K562, HepG2, HSMM, HUVEC, NHEK, and NHLF. For each variant, we investigated whether it is mapped to functional regions (i.e. promoter and enhancer) through chromatin states annotation from the UCSC Genome Browser (http://genome.ucsc.edu). The epigenetic landscape of histone markers H3K4Me1, H3K4Me3, and H3K27Ac was also examined through layered histone tracks on multiple ENCODE cell from the UCSC Genome Browser. DNase I hypersensitive and TF ChIP-Seq datasets were investigated in all available ENCODE cell lines. We evaluated each newly identified SNP whether it lies in a predicted motif based on RegulomeDB ^34^. Additionally, we evaluated those SNPs whether are eQTLs based on publically available database eQTL browser (http://eqtl.uchicago.edu/cgi-bin/gbrowse/eqtl/).

To examine whether the genes located near the newly- and previously-identified loci for WC or WHR converge to certain biological pathways, we examined their functional enrichment using the ingenuity pathway analysis (IPA) tool in Ingenuity (version 17199142) (http://www.ingenuity.com/products/ipa).

Reference

1. Gao, Y. T. *et al.* Association of menstrual and reproductive factors with breast cancer risk: results from the Shanghai Breast Cancer Study. *Int.J.Cancer* **87,** 295–300 (2000).

2. Zheng, W. *et al.* Genome-wide association study identifies a new breast cancer susceptibility locus at 6q25.1. *Nat.Genet.* **41,** 324–328 (2009).

3. Wen, W. *et al.* The modifying effect of C-reactive protein gene polymorphisms on the association between central obesity and endometrial cancer risk. *Cancer* **112,** 2409–2416 (2008).

4. Shu, X. O. *et al.* Soy food intake and breast cancer survival. *JAMA* **302,** 2437–2443 (2009).

5. Zheng, W. *et al.* The Shanghai Women’s Health Study: rationale, study design, and baseline characteristics. *Am.J.Epidemiol.* **162,** 1123–1131 (2005).

6. Shu, X.-O. *et al.* Cohort Profile: The Shanghai Men’s Health Study. *Int. J. Epidemiol.* doi:10.1093/ije/dyv013 (2015).

7. Tsai, F.-J. *et al.* A genome-wide association study identifies susceptibility variants for type 2 diabetes in Han Chinese. *PLoS Genet.* **6,2**; e1000847. doi: 10.1371/journal.pgen (2010).

8. Pan, W.-H. *et al.* Han Chinese cell and genome bank in Taiwan: purpose, design and ethical considerations. *Hum. Hered.* **61,** 27–30 (2006).

9. Lee, M. T. M. *et al.* Genome-wide association study of bipolar I disorder in the Han Chinese population. *Mol. Psychiatry* **16,** 548–556 (2011).

10. Ye, X. *et al.* Distributions of C-reactive protein and its association with metabolic syndrome in middle-aged and older Chinese people. *J.Am.Coll.Cardiol.* **49,** 1798–1805 (2007).

11. GenSalt Collaborative Research Group. GenSalt: rationale, design, methods and baseline characteristics of study participants. *J.Hum.Hypertens.* **21,** 639–646 (2007).

12. Purcell, S. *et al.* PLINK: a tool set for whole-genome association and population-based linkage analyses. *Am. J. Hum. Genet.* **81,** 559–575 (2007).

13. Abecasis, G. R., Cherny, S. S., Cookson, W. O. & Cardon, L. R. GRR: graphical representation of relationship errors. *Bioinforma. Oxf. Engl.* **17,** 742–743 (2001).

14. O’Connell, J. R. & Weeks, D. E. PedCheck: a program for identification of genotype incompatibilities in linkage analysis. *Am. J. Hum. Genet.* **63,** 259–266 (1998).

15. Wang, F. *et al.* Cohort profile: The Dongfeng-Tongji cohort study of retired workers. *Int.J.Epidemiol.* **42,**3,731-40. doi: 10.1093/ije/dys053 (2012).

16. Aulchenko, Y. S., Struchalin, M. V. & van Duijn, C. M. ProbABEL package for genome-wide association analysis of imputed data. *BMC.Bioinformatics.* **11**,134. doi: 10.1186/1471-2105-11-134. (2010).

17. Hughes, K. *et al.* Cardiovascular diseases in Chinese, Malays, and Indians in Singapore. II. Differences in risk factor levels. *J. Epidemiol. Community Health* **44,** 29–35 (1990).

18. Tan, C. E., Emmanuel, S. C., Tan, B. Y. & Jacob, E. Prevalence of diabetes and ethnic differences in cardiovascular risk factors. The 1992 Singapore National Health Survey. *Diabetes Care* **22,** 241–247 (1999).

19. Hughes, K., Aw, T. C., Kuperan, P. & Choo, M. Central obesity, insulin resistance, syndrome X, lipoprotein(a), and cardiovascular risk in Indians, Malays, and Chinese in Singapore. *JEpidemiolCommunity Health* **51,** 394–399 (1997).

20. Cutter, J., Tan, B. Y. & Chew, S. K. Levels of cardiovascular disease risk factors in Singapore following a national intervention programme. *BullWorld Health Organ* **79,** 908–915 (2001).

21. Marchini, J., Howie, B., Myers, S., McVean, G. & Donnelly, P. A new multipoint method for genome-wide association studies by imputation of genotypes. *Nat.Genet.* **39,** 906–913 (2007).

22. Chambers, J. C. *et al.* Common genetic variation near MC4R is associated with waist circumference and insulin resistance. *Nat.Genet.* **40,** 716–718 (2008).

23. Bild, D. E. *et al.* Multi-ethnic study of atherosclerosis: objectives and design. *Am.J.Epidemiol.* **156,** 871–881 (2002).

24. Cho, Y. S. *et al.* A large-scale genome-wide association study of Asian populations uncovers genetic factors influencing eight quantitative traits. *Nat.Genet.* **41,** 527–534 (2009).

25. Kim, Y. J. *et al.* Large-scale genome-wide association studies in East Asians identify new genetic loci influencing metabolic traits. *Nat. Genet.* **43,** 990–995 (2011).

26. Jee, S. H. *et al.* Adiponectin concentrations: a genome-wide association study. *Am.J.Hum.Genet.* **87,** 545–552 (2010).

27. Takeuchi, F. *et al.* Blood pressure and hypertension are associated with 7 loci in the Japanese population. *Circulation* **121,** 2302–2309 (2010).

28. Takeuchi, F. *et al.* Confirmation of multiple risk Loci and genetic impacts by a genome-wide association study of type 2 diabetes in the Japanese population. *Diabetes* **58,** 1690–1699 (2009).

29. Adair, L. S. *et al.* Cohort profile: the Cebu longitudinal health and nutrition survey. *Int.J.Epidemiol.* **40,** 619–625 (2011).

30. Lange, L. A. *et al.* Genome-wide association study of homocysteine levels in Filipinos provides evidence for CPS1 in women and a stronger MTHFR effect in young adults. *Hum.Mol.Genet.* **19,** 2050–2058 (2010).

31. Nanri, A. *et al.* Dietary patterns and C-reactive protein in Japanese men and women. *Am.J.Clin.Nutr.* **87,** 1488–1496 (2008).

32. Asano, H. *et al.* Plasma resistin concentration determined by common variants in the resistin gene and associated with metabolic traits in an aged Japanese population. *Diabetologia* **53,** 234–246 (2010).

33. Ward, L. D. & Kellis, M. HaploReg: a resource for exploring chromatin states, conservation, and regulatory motif alterations within sets of genetically linked variants. *Nucleic Acids Res.* **40,** D930–934 (2012).

34. Boyle, A. P. *et al.* Annotation of functional variation in personal genomes using RegulomeDB. *Genome Res* **22,** 1790–1797 (2012).
